# Clinical efficacy and mechanistic study of fulvning granules in symptomatic atrial fibrillation: a randomized controlled trial with untargeted metabolomics analysis

**DOI:** 10.3389/fphar.2026.1761563

**Published:** 2026-02-24

**Authors:** Jinru Tao, Keke Liu, Tianyi Cheng, Mianmian Li, Chunrui Hu, Yulai Zhan, Qiuting Yu, Yinyifan Zhou, Xue Liu, Zilin Ma, Na Zhang, Bing Deng, Lin Shen, Nuo Tang, Qiong Wu

**Affiliations:** 1 Department of Cardiology, Longhua Hospital, Shanghai University of Traditional Chinese Medicine, Shanghai, China; 2 Department of Traditional Chinese Medicine, Yangpu Hospital, Tongji University, Shanghai, China; 3 Shanghai University of Traditional Chinese Medicine, Shanghai, China

**Keywords:** clinical trial, fulvning granules, metabolomics analysis, symptomatic atrial fibrillation, traditional Chinese medicine

## Abstract

**Background:**

Atrial fibrillation (AF) is the most prevalent sustained arrhythmia and a leading cause of morbidity and mortality worldwide. Although antiarrhythmic drugs and catheter ablation have improved AF management, their therapeutic efficacy remains suboptimal. Fulvning Granules (FLN), a regulated hospital preparation officially approved by the Shanghai Drug Administration, have shown promising clinical efficacy in local practice. However, robust high-level clinical evidence is required to validate their benefits and elucidate the underlying mechanisms.

**Materials and methods:**

A randomized, double-blind, placebo-controlled trial enrolled 136 symptomatic AF patients, who received either FLN or a placebo for 4 weeks in addition to standard guideline-directed medical therapy (GDMT). The primary endpoint was AF control effectiveness, assessed by 24-h Holter monitoring. Secondary endpoints included palpitation frequency and duration, echocardiographic evaluation of cardiac structure and function, N-terminal pro-B-type natriuretic peptide (NT-pro BNP)levels, Hamilton Anxiety (HAMA) and Depression (HAMD) Scales, and the 36-item Short-Form Health Survey (SF-36) from baseline to week 4. To further validate FLN’s efficacy and explore its mechanisms, serum-based metabolic pathway analysis was conducted to investigate the metabolic network associated with FLN treatment of AF.

**Results:**

FLN significantly improved AF control compared with placebo (78.57% vs. 54.39%; *P* = 0.001), with concomitant reductions in both the frequency and duration of palpitations. In addition, treatment with FLN markedly enhanced psychological well-being and health-related quality of life. Untargeted metabolomics analysis identified 23 bioactive compounds in FLN and revealed significant modulation of ketone, butanoate, and glycerophospholipid pathways (*P* < 0.05), primarily involving acylcarnitines, Lutein, and LysoPC (22:0).

**Conclusion:**

FLN serves as a safe and effective adjuvant therapy for reducing AF episode frequency and ventricular rate in patients with symptomatic AF. Its mechanism may involve the modulation of cardiac energy metabolism.

**Clinical Trial Registration:**

ClinicalTrials.gov, identifier ChiCTR2000036835.

## 1 Introduction

Atrial fibrillation is the most prevalent sustained arrhythmia and a major contributor to global morbidity and mortality (Joglar et al., 2024). Epidemiological data suggest that AF burden in the Asia-Pacific region is projected to surpass that of North America and Europe, with the number of AF patients expected to reach 72 million by 2050 (Lippi et al., 2021). As a major population center within the Asia-Pacific, China has seen a twentyfold increase in AF prevalence over the past decade, reaching 1.6% in 2015 (Shi et al., 2022). AF worsens quality of life (QoL) and increases the risk of stroke, heart failure (HF), arrhythmic events, and mortality. Its incidence rises with aging, cardiac dysfunction, comorbidities, and poor lifestyle habits, leading to longer hospital stays, higher costs, and worse outcomes (Kirchhof et al., 2020; Hindricks et al., 2021).

Current AF treatments include pharmacological therapy, catheter ablation, and left atrial appendage occlusion. Pharmacological management primarily consists of antiarrhythmic drugs (AADs) for rhythm or rate control, along with anticoagulants. While pharmacological cardioversion has a success rate of 58%–75%, significant variability and adverse effects limit its widespread use (Dixit et al., 2024; Valembois et al., 2019). Catheter ablation is an effective rhythm-control strategy for drug-refractory symptomatic paroxysmal AF, supported by multiple randomized trials and large registries (Joglar et al., 2024; Schnabel et al., 2023). However, 30%–40% of patients experience recurrence after the first procedure, with recurrence rates up to 45.9% within a year (Joglar et al., 2024). Additionally, thermal injury from radiofrequency ablation can lead to intraoperative pain and postoperative complications, affecting procedural success and patient outcomes. Given the importance of patient selection in determining ablation efficacy (Iliodromitis et al., 2023), it cannot be considered a first-line treatment for all patients, highlighting the need for novel strategies to improve long-term rhythm control, reduce recurrence, and enhance outcomes.

Traditional Chinese Medicine (TCM) offers distinct advantages in treating cardiovascular diseases through its multi-metabolite, multi-target approach. Growing evidence confirms the antiarrhythmic properties of various herbal formulations (Huang et al., 2024; Quan et al., 2023). FLN is a sophisticated hospital preparation developed by Professor Zhongxiang Lin based on decades of specialized clinical experience in arrhythmia management at Longhua Hospital. It is a modified formulation derived from two landmark classical TCM prescriptions—Shengmai San (for nourishing Qi and Yin) and Ganmai Dazao Tang (for emotional regulation and spirit-calming) (National Pharmacopoeia Committee, 2020). Approved for market in China in 2004 (Approval Number: Z20030058), FLN has demonstrated comparable efficacy to propafenone in treating Qi and Yin deficiency-type arrhythmias (Liu et al., 2017; Wei et al., 2017). Clinical studies also report improvements in TCM-related symptoms with a favorable safety profile. A recent study also showed that FLN can reduce the frequency and duration of symptomatic atrial fibrillation episodes and help control ventricular rate (Liu et al., 2023). However, due to limited sample sizes and a lack of robust clinical trials, further investigation is needed.

Metabolomics, as a part of systems biology, is an approach that involves the comprehensive analysis of all metabolites during a specific physiological period (Qiu et al., 2023). With the continuous development of metabolomics, an increasing number of metabolites have been discovered and studied, providing new insights for a deeper understanding of the mechanisms underlying cardiovascular diseases (Lv et al., 2024). This study integrated plasma metabolomics analysis to further explore the mechanism of FLN in treating symptomatic AF. To our knowledge, this is the first randomized double-blind placebo-controlled clinical study combining metabolomics to investigate TCM treatment of symptomatic AF. It provides a new research strategy for exploring the efficacy of TCM and offers scientific reference for understanding the etiology and pathogenesis of Qi-Yin deficiency syndrome in AF treatment with FLN.

## 2 Materials and methods

### 2.1 Study design and settings

This study was a prospective, randomized, double-blind, placebo-controlled clinical trial conducted at Longhua Hospital, affiliated with the Shanghai University of TCM. The study was approved by the Institutional Review Board of Longhua Hospital (No. 2021LCSY051) and conducted in accordance with the principles of Good Clinical Practice (GCP) and the Declaration of Helsinki. The trial was registered in the Chinese Clinical Trials Registry on 20 September 2020 (ChiCTR2000036835).

Investigational product: FLN is a regulated Hospital Preparation officially approved by the Shanghai Drug Administration (Approval No. Z2003005). The granules were manufactured and supplied by Shanghai Baolong Pharmaceutical Co., Ltd. (Shanghai, China) in compliance with Good Manufacturing Practice (GMP) standards. The product for this study consisted of a single manufacturing batch (Batch No. 2103001) to ensure consistency. FLN is composed of 15 TCM substances, including 13 botanical drugs and 2 mineral substances. The detailed composition, including taxonomic validation against Plants of the World Online (POWO), medicinal parts, and daily dosages, is listed in Table 1.

**TABLE 1:** Composition of fulvning granules (FLN).

No.	Chinese name	Botanical/Scientific name (Latin binomial)	Family	Drug name (pharmacopoeial)	Daily dose (g)
1	Dang Shen	*Codonopsis pilosula* (Franch.) Nannf.	Campanulaceae	Codonopsis Radix	15
2	Mai Dong	*Ophiopogon japonicus* (Thunb.) Ker Gawl.	Asparagaceae	Ophiopogonis Radix	12
3	Wu Wei Zi	*Schisandra chinensis* (Turcz.) Baill.	Schisandraceae	Schisandrae Chinensis Fructus	9
4	Huai Xiao Mai	*Triticum aestivum* L.	Poaceae	Tritici Levis Fructus	30
5	Zhi Gan Cao	*Glycyrrhiza uralensis* Fisch. ex DC.	Fabaceae	Glycyrrhizae Radix et Rhizoma Praeparata	6
6	Da Zao	*Ziziphus jujuba* Mill.	Rhamnaceae	Jujubae Fructus	9
7	Tu Si Zi	*Cuscuta chinensis* Lam.	Convolvulaceae	Cuscutae Semen	15
8	Gou Qi Zi	*Lycium barbarum* L.	Solanaceae	Lycii Fructus	15
9	Long Gu	*Fossilia Ossis Mastodi* (Mineral/Fossil)	N/A	Os Draconis	30
10	Mu Li	*Crassostrea gigas* (Thunb.)	Ostreidae	Ostreae Concha	30
11	Dan Shen	*Salvia miltiorrhiza* Bunge	Lamiaceae	Salviae Miltiorrhizae Radix et Rhizoma	15
12	Huang Qin	*Scutellaria baicalensis* Georgi	Lamiaceae	Scutellariae Radix	30
13	Ku Shen	*Sophora flavescens* Aiton	Fabaceae	Sophorae Flavescentis Radix	15
14	Cha Shu Gen	*Camellia sinensis* (L.) Kuntze	Theaceae	Camelliae Radix	15
15	Gua Lou Pi	*Trichosanthes kirilowii* Maxim.	Cucurbitaceae	Trichosanthis Pericarpium	20

Compliance with ethical and environmental standards:

None of the ingredients in FLN are derived from endangered species listed in the CITES Appendices or the IUCN Red List. All raw materials were ethically sourced from GAP-certified cultivation bases.

### 2.2 Participants

AF diagnosis criteria were based on 2020 ESC Guidelines for the diagnosis and management of atrial fibrillation (Hindricks et al., 2021). Eligible participants were aged 18 to 85, with at least one electrocardiogram (ECG) confirming AF and presenting clear symptoms of palpitations. The detailed inclusion and exclusion criteria were as follows.

Inclusion criteria: Inclusion criteria: (1) Aged 18–85 years; (2) Diagnosed with paroxysmal AF (≥2 episodes/month) or permanent AF, confirmed by at least one ECG; (3) Clear symptoms of palpitations; (4) Meets TCM syndrome diagnostic criteria; (5) Understands the study’s purpose, adheres to research protocols, and voluntarily provides informed consent.

Exclusion criteria: (1) Severe primary diseases, including lung, liver, kidney, hematopoietic dysfunction, or severe heart dysfunction (NYHA Class IV); (2) AF with identifiable causes such as fatigue, mental stress, emotional fluctuations, drug toxicity, or electrolyte imbalance; (3) Mental illness or poorly controlled psychiatric disorders; (4) Heart rate <50 bpm due to conditions like sick sinus syndrome, atrioventricular block, or intraventricular conduction block (including those requiring a pacemaker); (5) Pregnant or lactating women; (6) Cachexia due to terminal-stage malignant tumors; (7) Participation in other TCM clinical studies in the past 3 months or unwillingness to discontinue other TCM treatments; (8) Inability to assess efficacy due to incomplete data or other factors. (9) Known allergy or hypersensitivity to any of the botanical drugs or excipients contained in the FLN formulation.

### 2.3 Randomization and masking

#### 2.3.1 Randomization

This study uses a center-based stratification and block randomization method. The randomization sequence will be generated by study investigators who are statisticians. Patients will be allocated in a 1:1 ratio, aiming to balance baseline characteristics between the groups. Participants will be assigned a PID number, which will be used for subject identification throughout the study. Information regarding the random-number block will be delivered to the participating centers along with the intervention drugs.

#### 2.3.2 Double-blind

The study is designed as a double-blind investigation. The participants, study monitors, and study investigators will be blinded throughout the duration of the study. The PID will be the only information linked to group allocation. Random codes will be maintained by Xuejun Cui, associate researcher, and director of the Office of National Traditional Chinese Medicine Clinical Research Base of Longhua Hospital to ensure concealment.

### 2.4 Sample size

The sample size of this study was calculated based on the analysis of data published in previous articles on the treatment of AF by Chinese medicine and the expected value of efficacy. It was assumed that the total efficacy rates in the study and control groups were 90.7% and 70.27%, respectively (HE et al., 2018). Thus, assuming α = 0.05 and power = 0.8, the calculated sample size required for each group is 54 cases, after substituting the above values in PASS 15 Considering that 20% will miss visits, the sample size is adjusted to 68 cases in each group, and a total of 136 cases.

### 2.5 Screening and group assignment

All patients provided written informed consent before screening and enrollment. After meeting the initial criteria, participants entered a screening period (−7 to 0 days). Eligible patients were randomly assigned in a 1:1 ratio to the treatment group (FLN combined with standard western medicine) or the control group (Placebo combined with standard western medicine) for 4 weeks.

Throughout the trial, all participants continued to receive GDMT, including anticoagulants, antiarrhythmic drugs, and myocardial energy metabolism drugs, strictly in accordance with the 2020 ESC Guidelines for the Diagnosis and Management of Atrial Fibrillation. To ensure the reliability of efficacy evaluation, patients were required to be on a stable regimen of these standard medical therapies for at least 2 weeks prior to enrollment. The dosage of these background medications was kept stable during the intervention period unless a safety emergency required adjustment.

### 2.6 Interventions

The FLN and placebo were produced and packed in a single batch (production batch number: FLN: 2103001; Placebo: 2103001) by Shanghai Baolong Pharmaceutical Co., Ltd., which has no conflicts of interest relevant to this study. The placebo is composed of 10% crude FLN and 90% starch, which have the same appearance and scent as the active treatment drugs. Participants took one bag of FLN or Placebo twice a day for 4 weeks as an adjuvant therapy to their ongoing standard medical treatment. Concurrent use of other TCM was prohibited to avoid confounding effects.

Chemical Profiling and Quality Control of FLN: The chemical composition of FLN was characterized using UPLC-Q-TOF-MS (Waters H-Class combined with AB Sciex Triple TOF 4600). Chromatographic separation was achieved on a Welch Ultimate AQ-C18 column (100 mm × 2.1 mm, 1.8 μm) using an acetonitrile and 0.1% formic acid gradient. Mass spectrometry was operated in both ESI+ and ESI- modes (m/z 50–1,500). Compounds were identified by matching accurate mass (error < 5 ppm) and MS/MS fragments against the Standard Natural Product HR-MS Database and literature. A total of 23 major compounds (e.g., Matrine, Baicalin, and Salvianolic acid B) were identified. Detailed analytical conditions, the annotated Total Ion Chromatogram (TIC), and the compound list are provided in Supplementary Figure S1, and Supplementary Tables S1, S2.

### 2.7 Outcome

#### 2.7.1 Primary outcome

A 24-h Holter monitor was used to assess cardiac electrical activity, including the total duration and proportion of time in AF, episodes of ectopic atrial tachycardia, total heartbeats over 24 h, and the average ventricular rate before and after treatment. The efficacy evaluation criteria are as follows.

##### 2.7.1.1 Paroxysmal AF

Clinical Control: No atrial fibrillation episodes confirmed by instrumental examination during treatment.

Markedly Effective: Atrial fibrillation episodes occur during treatment, but the frequency decreases by ≥70% compared to pre-treatment.

Effective: Atrial fibrillation episodes occur during treatment, but the frequency decreases by ≥50% and <70% compared to pre-treatment.

Ineffective: Atrial fibrillation episodes decrease by <50% or increase compared to pre-treatment.

##### 2.7.1.2 Permanent AF

Significant Effect: 24-h average ventricular rate of 60–80 beats/min or a reduction of ≥20 beats/min.

Effective: 24-h average ventricular rate of 81–90 beats/min or a reduction of <20 beats/min.

Ineffective: No reduction or an increase in ventricular rate.

#### 2.7.2 Secondary outcomes

##### 2.7.2.1 Frequency and duration of heart palpitation episodes

Each participant received a pre-designed booklet to record the frequency and duration of heart palpitations. Comprehensive instructions were provided to ensure accurate and consistent self-recording.

##### 2.7.2.2 Cardiac structure and function

Echocardiography assessed left atrial diameter (LAD), left ventricular end-diastolic diameter (LVEDD), left ventricular stroke volume (LVSV), and ejection fraction (LVEF). NT-pro BNP levels were measured as a sensitive biomarker of heart failure, which is commonly associated with atrial fibrillation.

##### 2.7.2.3 HAMA, HAMD and SF-36

HAMA and HAMD were used to assess the severity of anxiety and depression, respectively, while SF-36 measured patients’ quality of life.

##### 2.7.2.4 Metabolomic profiling of serum samples using LC-MS/MS

Whole blood was collected from patients using EDTA anticoagulant tubes and centrifuged at 3,000 rpm for 10 min at room temperature within 1 h of collection. The supernatant was aliquoted into 1.5 mL centrifuge tubes at 0.2 mL per tube and stored at −80 °C. Samples were shipped on dry ice. Metabolomic analysis was conducted by Shanghai Biotree Biomedical Technology Co., Ltd. (BIOTREE).

#### 2.7.3 Adverse events

Safety monitoring was conducted throughout the study. Adverse events (AEs) were recorded and managed according to clinical judgment. Serious adverse events (SAEs) were reported to the institutional ethics committee in accordance with local regulations. Safety evaluation included routine blood tests, liver and kidney function tests, electrolyte analysis, and urinalysis.

### 2.8 Data management and monitoring

Data were collected and managed using the Redcap electronic data capture tools hosted on the Longhua Hospital platform. The Shenkang Center ensures data authenticity, traceability, and security through integration with the CRIP data platform. CRAs oversee study management, including researcher qualifications, project ethics, data integrity, and adverse event reporting. Paper-based Case Report Forms (CRFs) are maintained for informed consent and interview scales. Personal medical records are accessible only to the investigator, who signs a confidentiality agreement. Data are processed anonymously, with any identifiable information omitted. The project management group meets monthly to review trial conduct, while the GCP office and ethics committee meet biannually. The Shenkang Center conducts an annual audit to verify data consistency and ensure compliance with protocols. Unauthorized changes are deemed invalid.

### 2.9 Statistical analysis

The full analysis set (FAS) was used for baseline characteristics analysis, while the per-protocol set (PPS) was used for all primary and secondary efficacy endpoint analyses. Safety analyses were conducted using the safety set (SS). Continuous variables were presented as means ± standard deviations (SDs) or medians with interquartile ranges (IQRs). All data were exported from the REDCap electronic CRF system and analyzed using SPSS 24.0 statistical software. For measurement data that follow a normal distribution, t-tests were performed, and results were expressed as mean ± SD. For data that do not follow a normal distribution, anon-parametric test (rank-sum test) was used, with results presented as median (IQR). The χ^2^ test was used for categorical data, and the rank-sum test was applied for ordered categorical data. A two-sided test was used, with *P* > 0.05 indicating no statistical significance and *P* < 0.05 indicating statistical significance.

## 3 Results

### 3.1 Participants and baseline characteristics

**FIGURE 1:**
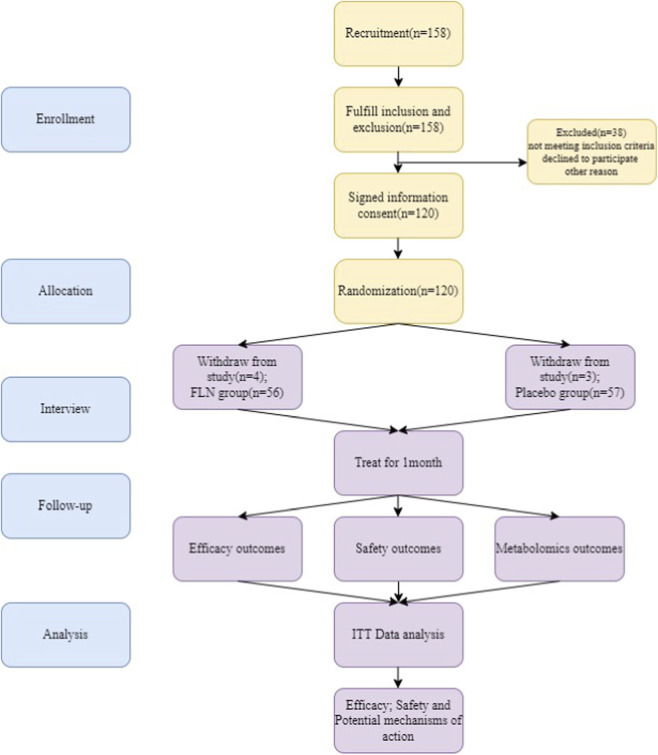
The flow chart of the clinical procedures through the study.

From 25 March 2021 through 2 March 2023, a total of 168 patients were invited to participate, 32 patients either did not respond or did not meet the eligibility criteria. The remaining 136 participants completed the initial eligibility assessment and were randomly assigned to either the FLN group (n = 68) or the placebo group (n = 68). During the study, 23 participants were lost to follow-up due to reasons such as pandemic-related quarantine measures or long travel distances, resulting in 113 participants successfully completing the intervention, which remained within a controllable and acceptable range (Figure 1). Among them, 56 were in the FLN group and 57 participants were in the placebo group. A total of 72 participants had paroxysmal AF (37 in the placebo group and 35 in the treatment group), while 41 participants had permanent AF (20 in the placebo group and 21 in the treatment group) (Table 1).

Comparison of the FLN and placebo groups showed no significant differences at baseline in demographic characteristics, clinical characteristics and concomitant use of therapies (*P* > 0.05) (Table 2). Thus, the baseline characteristics of the FLN and placebo groups in this study were balanced and comparable.

**TABLE 2:** Baseline characteristics.

Parameters		FLN (n = 56)	Placebo (n = 55)	*P-*value
Demographics				
Type of AF (n (%))	Paroxysmal AF	35	37	0.790
	Permanent AF	21	20	
Age (years)		69.50 (65.00,77.00)	70.00 (64.00,76.00)	0.944
Male sex (n (%))		27 (48.2)	31 (56.4)	0.512
Body mass index^a^(kg m^-2^)		22.68 (20.93,24.40)	23.15 (21.23,25.10)	0.185
Tympanic temperature (°C)		36.80 (36.80,36.90)	36.70 (36.60,36.80)	0.380
Systolic BP(mmHg)		124.50 (118.00,126.50)	125.00 (118.00,127.00)	0.526
Diastolic BP(mmHg)		75.00 (75.00,78.00)	75.00 (75.00,78.00)	0.959
Resting heart rate (beats/min)		79.00 (72.00,95.00)	85.00 (75.00,98.00)	0.257
Resting respiratory rate (beats/min)		19.00 (19.00,19.00)	19.00 (19.00,19.00)	0.340
Disease duration (months)		30.00 (6.25,72.00)	24.00 (12.00,60.00)	0.444
Smoking (n (%))		18 (32.1)	22 (40.0)	0.389
Alcohol consumption		22 (39.3)	26 (47.3)	0.396
CHA_2_DS_2_-VASc score		2.50 (2.00,4.00)	2.00 (2.00,3.00)	0.998
HAS-BLED score		1.00 (1.00,2.00)	1.00 (1.00,2.00)	0.797
ECG ventricular rate (beats/min)		89.00 (70.25,113.25)	91.00 (76.00,107.00)	0.695
Comorbidities				
Hypertension (n (%))		35 (62.5)	30 (54.5)	0.395
Coronary heart disease		12 (21.4)	10 (18.2)	0.668
Diabetes mellitus		10 (17.9)	11 (20.0)	0.773
Hyperlipidemia		9 (16.1)	11 (20.0)	0.590
Valvular disease		3 (5.4)	1 (1.8)	0.623
Myocarditis		0 (0.0)	2 (3.6)	0.468
Cardiomyopathy		2 (3.6)	0 (0.0)	0.483
Concomitant therapy				
Warfarin		10	7	0.453
Dabigatran		2	4	0.658
Rivaroxaban		26	28	0.637
Edoxaban		4	4	1.000
Propafenone		4	6	0.718
Amiodarone		8	6	0.592
Sotalol		2	3	0.984
Bisoprolol		5	8	0.358
Metoprolol succinate		22	23	0.235

^a^ The body mass index was calculated as weight (kg) divided by the square of height (m).

### 3.2 Primary outcomes

**FIGURE 2:**
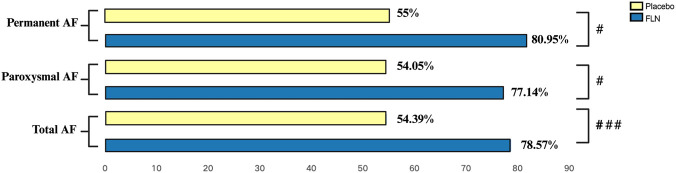
Effective Control Rates in Patients With Atrial Fibrillation. Comparison between groups: ^#^ *P* < 0.05 and ^###^ *P* < 0.001.

The primary endpoint was the AF control rate, assessed by 24-h Holter monitoring. As shown in Figure 2, AF was effectively controlled in 78.57% of patients receiving FLN, compared to 54.39% in the control group, with a statistically significant difference (*P* = 0.001). Among patients with paroxysmal AF, the control rates were 77.14% with FLN and 55.00% in the control group (*P* = 0.018), while in those with permanent AF, the rates were 80.95% and 61.11%, respectively (*P* = 0.035). These findings suggest that the therapeutic effect of FLN was observed in both paroxysmal and permanent AF (Figure 2).

**FIGURE 3:**
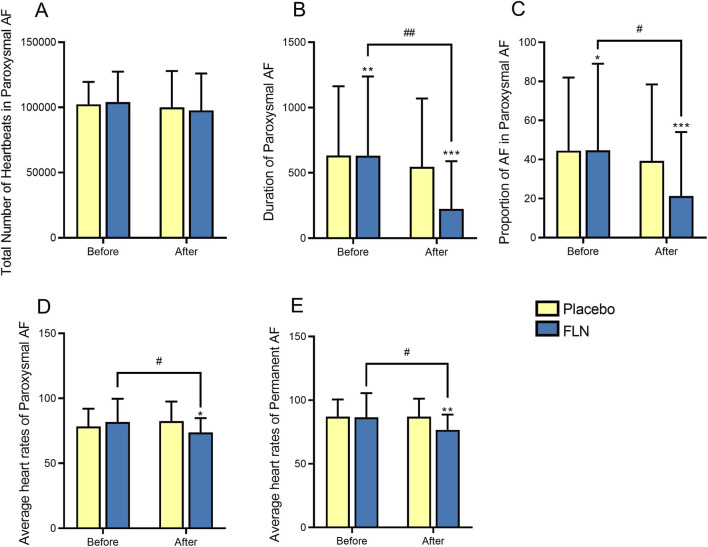
Comparison of Holter Data. **(A)** Total Number of Heartbeats in Paroxysmal AF; **(B)** Duration of Paroxysmal AF; **(C)** Proportion of AF in Paroxysmal AF; **(D)** Average heart rates of Paroxysmal AF; **(E)** Average heart rates of Permanent AF. Comparison within group: ^*^P < 0.05, ^**^ *P* < 0.01 and ^***^ *P* < 0.001; Comparison between groups: ^#^ *P* < 0.05 and ^##^ *P* < 0.01.

To better understand which specific Holter parameters contributed to these improvements, we analyzed key electrophysiological data. Holter monitoring revealed that in patients with paroxysmal AF, the total number of beats did not differ significantly within or between groups (*P* > 0.05). However, AF duration, the average proportion of AF episodes, and mean heart rate were significantly improved (*P* < 0.05), with the treatment group demonstrating greater reductions in AF duration and episode proportion compared to the control group. In patients with permanent AF, the mean heart rate recorded by Holter after treatment showed a significant intergroup difference (*P* < 0.05), suggesting that FLN was more effective than placebo in lowering heart rate (Figure 3).

### 3.3 Secondary outcomes

#### 3.3.1 Frequency and duration of heart palpitation episodes

After treatment, comparisons between the two groups showed FLN significantly reduced both the frequency (*P* = 0.035) and duration (*P* = 0.047) (Table 3) of palpitations compared to the placebo group. Specifically, the FLN group showed a greater reduction in both the frequency and duration of palpitations, with these differences being statistically significant (*P* < 0.01). These findings indicate that FLN is more effective than the placebo in reducing both the frequency and duration of palpitations in patients with AF.

**TABLE 3:** Comparison of palpitation in paroxysmal AF [M (Q1, Q3)] (times/month, min/month).

Index	Group	Pre-treatment	After treatment	within a group		intergroup	
				Z	*P -*value	Z	*P-*value
Number of palpitation episodes	Placebo	5.00 (3.00,6.00)	4.00 (2.00,6.00)	−1.934	0.053	−2.110	0.035^#^
	FLN	5.00 (4.00,8.00)	3.00 (0.00,5.00)	−4.953	0.000^***^		
Palpitation duration	Placebo	60.00 (30.00,120.00)	55.00 (22.50,120.00)	−0.989	0.323	−1.983	0.047^#^
	FLN	60.00 (30.00,120.00)	20.00 (0.00,75.00)	−5.021	0.000^***^		

Comparison within group: ^*^ *P* < 0.05, ^**^ *P* < 0.01 and ^***^ *P* < 0.001; Comparison between groups: ^#^ *P* < 0.05 and ^##^ *P* < 0.01.

#### 3.3.2 Cardiac function and biomarkers

**FIGURE 4:**
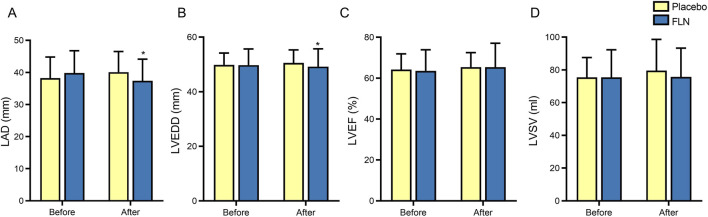
Analysis of cardiac ultrasound indicators. **(A)** LAD; **(B)** LVEDD; **(C)** LVEF; **(D)** LVSV. Comparison within group: ^*^ *P* < 0.05.

We assessed cardiac structure through echocardiography (LAD, LVEDD, LVSV) and cardiac function using both echocardiographic LVEF and NT-pro BNP levels before and after treatment. The study found no significant differences between the FLN and placebo groups (*P* > 0.05). Both intergroup echocardiographic parameters (Figure 4) and NT-pro BNP levels (Table 4) were comparable.

**TABLE 4:** Comparison of NT-proBNP in AF [M (Q1, Q3)].

Research indicators	time	FLN (n = 56)	Placebo (n = 55)	*P -*value
NT-proBNP	Pre-treatment	554.50 (246.00,1157.50)	747.00 (302.00,1270.00)	0.434
	After-treatment	656.00 (184.75,1827.50)	750.00 (231.00,1260.00)	0.737

#### 3.3.3 Impact of treatment on anxiety, depression, and quality of life in AF patients

**FIGURE 5:**
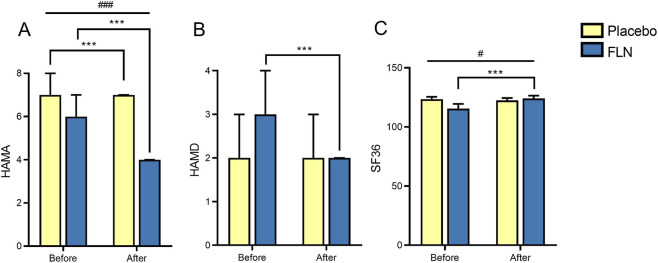
Indicator analysis of Hamilton Anxiety, Depression Scale, SF-36 Quality of Life Scale in AF. **(A)** HAMA; **(B)** HAMD; **(C)** SF-36.

After treatment, comparisons between the two groups of AF patients showed no significant statistical differences in HAMA, HAMD, or SF-36 (Figure 5), although a trend toward greater improvement was observed in the treatment group. To clarify this finding, we analyzed patients with paroxysmal and permanent AF separately. In paroxysmal AF, improvements in HAMA, HAMD, and SF-36 were significantly greater in the treatment group than in the control group (*P* < 0.05). In permanent AF, while HAMA and HAMD showed no statistical differences, the treatment group demonstrated significantly greater improvement in SF-36 compared with the control group (*P* < 0.05) (Table 5).

**TABLE 5:** Comparison of HAMA, HAMD and SF-36 [M(Q1,Q3)].

Index	Type of AF	Group	Pre-treatment	After treatment	within a group		intergroup	
					Z	P	Z	P
HAMA	Paroxysmal AF	Placebo	6.00 (5.00,8.00)	5.00 (4.00,6.00)	−3.563	0.000^***^	−2.109	0.035^#^
		FLN	6.00 (4.00,8.00)	4.00 (3.00,6.00)	−4.229	0.000^***^		
	Permanent AF	Placebo	4.50 (2.75,6.25)	4.50 (1.50,5.00)	2.179	0.044^*^	−1.298	0.194
		FLN	6.00 (4.00,7.50)	5.00 (4.00,7.00)	−2.085	0.037^*^		
HAMD	Paroxysmal AF	Placebo	2.00 (1.00,3.50)	2.00 (1.00,3.50)	−0.684	0.494	−2.015	0.044^#^
		FLN	3.00 (1.00,4.00)	2.00 (1.00,3.00)	−3.255	0.001^**^		
	Permanent AF	Placebo	1.50 (1.00,2.25)	1.50 (0.00,3.00)	−0.052	0.959	−1.231	0.218
		FLN	2.00 (1.50,4.00)	2.00 (1.00,4.00)	−0.525	0.599		
SF-36	Paroxysmal AF	Placebo	119.20 (111.40,123.90)	123.40 (112.90,127.20)	−2.281	0.029^*^	−2.104	0.035^#^
		FLN	117.40 (111.40,124.40)	124.40 (122.40,129.00)	−4.738	0.000^***^		
	Permanent AF	Placebo	122.00 (115.40,125.50)	123.20 (116.40, 124.80)	−0.600	0.549	0.038	0.031^#^
		FLN	120.00 (113.40,124.40)	124.40 (120.90,129.70)	−4.630	0.000^***^		

Comparison within group: ^*^ *P* < 0.05, ^**^ *P* < 0.01 and ^***^ *P* < 0.001; Comparison between groups: ^#^ *P* < 0.05.

#### 3.3.4 Metabolic profiling of plasma

Plasma samples from AF patients before and after oral administration of FLN were analyzed using metabolomics. Samples were classified as treat-F (pre-treatment) and treat-L (post-treatment), with 21 biological replicates per group, yielding a total of 42 samples.

**FIGURE 6:**
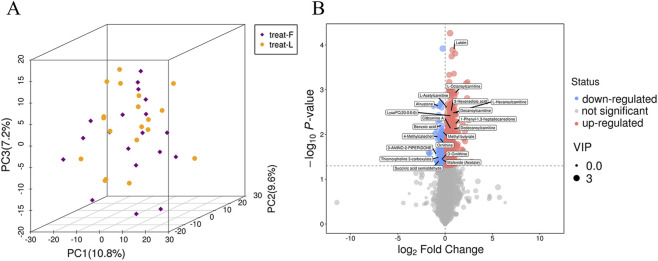
Basic data analysis. **(A)** 3D PCA scores between treat-F vs. treat-L. **(B)** Volcano plot between treat-F vs. treat-L. treat-F = pre-FLN; treat-L = post-FLN.

Experimental data were preprocessed and subjected to principal metabolite analysis (PCA) to assess overall metabolic differences. The three-dimensional PCA score plot (Figure 6A) showed that most samples fell within the 95% confidence interval, suggesting similar metabolite types and levels, with minimal overall metabolic variation.

**FIGURE 7:**
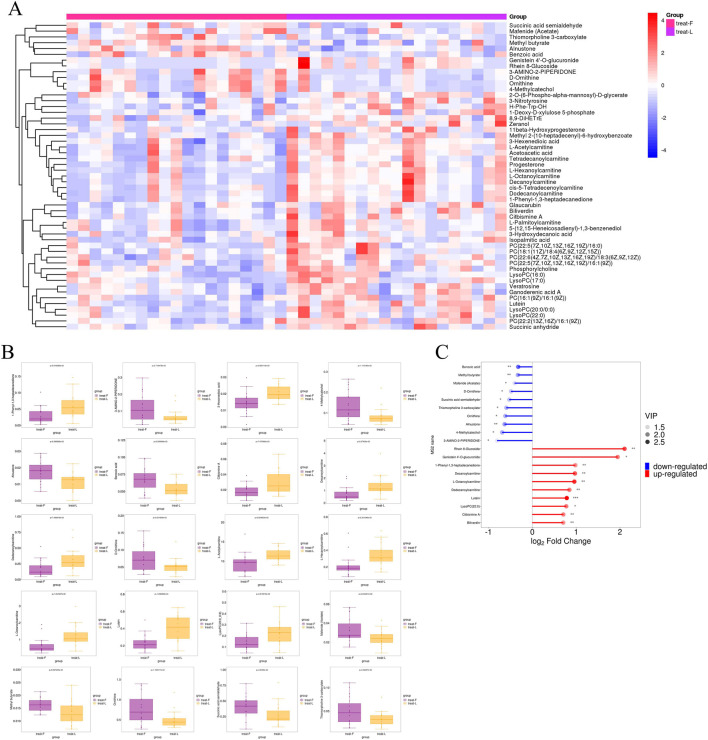
Untargeted Metabolomics. **(A)** Heatmap of hierarchical clustering analysis for group treat-F vs. treat-L; **(B)** Boxplot analysis for group treat-F vs. treat-L; **(C)** Matchstick analysis comparing treat-F and treat-L groups displayed the top 10 up- and downregulated metabolites ranked by fold-change. treat-F = pre-FLN; treat-L = post-FLN. ^*^0.01 < *P* < 0.05, ^**^ 0.001 < *P* < 0.01, ^***^ *P* < 0.001.

Differential metabolites were identified through univariate statistical analysis. A total of 8,720 metabolites were detected across both groups, with 687 showing significant differences—570 upregulated and 117 downregulated. Volcano plots (Figure 6B) visualized the overall distribution of these differential metabolites. Further hierarchical clustering analysis, incorporating p-values and fold changes, was performed to highlight significant differences (Figures 7A–C). Among the upregulated metabolites, Lutein exhibited the most significant increase (*P* < 0.001), while Biliverdin, Citbismine A, LysoPC (22:0), Dodecanoylcarnitine, L-Octanoylcarnitine, Decanoylcarnitine, 1-Phenyl-1,3-heptadecanedione, Genistein 4′-O-glucuronide, and Rhein 8-Glucoside also showed significant upregulation (*P* < 0.05). Conversely, Benzoic acid, Methyl butyrate, and Alnustone were among the most significantly downregulated metabolites (0.001 < *P* < 0.01), alongside 3-Amino-2-piperidone, 4-Methylcatechol, Ornithine, Thiomorpholine-3-carboxylate, Succinic acid semialdehyde, D-Ornithine, Mafenide (Acetate), and Methyl butyrate (*P* < 0.05).

**FIGURE 8:**
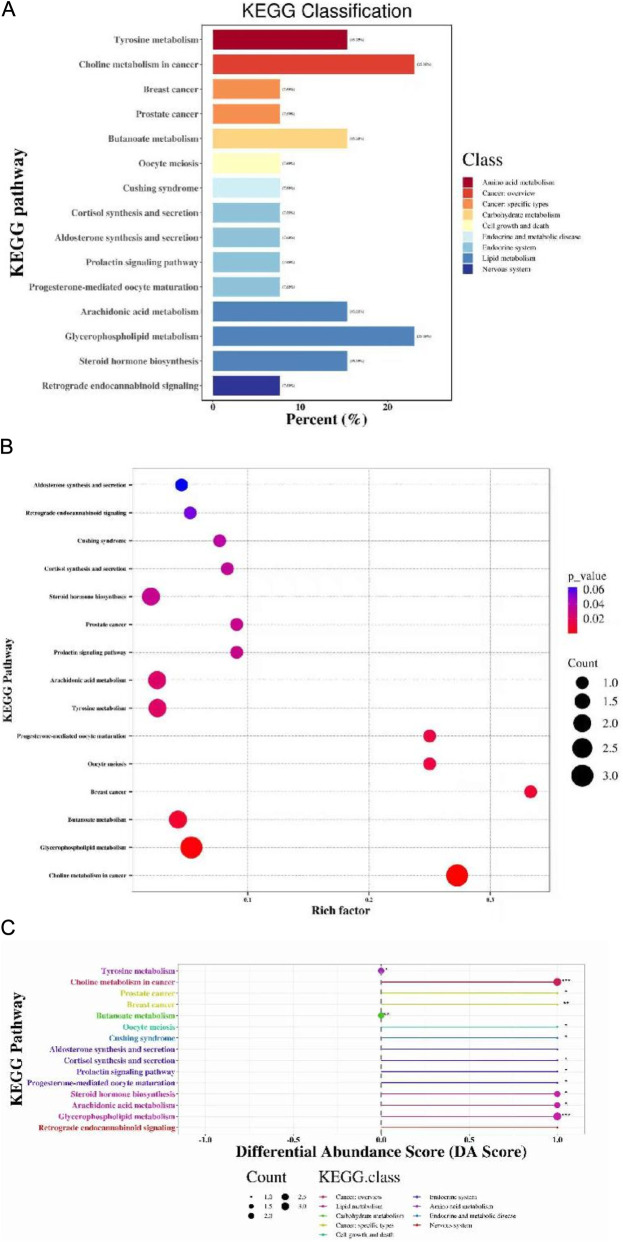
KEGG enrichment analysis of differential metabolites. **(A)** KEGG Classification for group treat-F vs. treat-L; **(B)** KEGG Enrichment for group treat-F vs. treat-L; **(C)** Differential Abundance Score for group treat-F vs. treat-L.

KEGG enrichment analysis was performed to classify differential metabolites (Figure 8A), assess their pathway enrichment (Figure 8B), and analyze their differential abundance (Figure 8C). A total of 15 KEGG pathways were identified. Notably, Glycerophospholipid metabolism (lipid metabolism) and Choline metabolism in cancer (cancer overview) exhibited the highest proportion of annotated differential metabolites (23.08%). Both pathways showed significant enrichment (*P* < 0.02), with all associated differential metabolites being upregulated.

**FIGURE 9:**
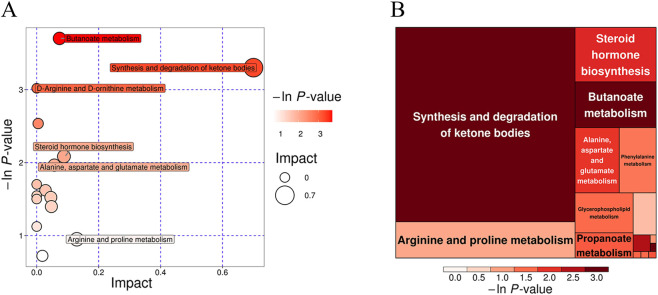
Metabolic pathway analysis of differential metabolites. **(A,B)** Pathway analysis for group treat-F vs. treat-L. The significance of enrichment is represented by -ln(*P*) values, where larger values/darker colors indicate a higher degree of statistical significance (ln *P* > 2.99 corresponds to *P* < 0.05).

After comprehensive analysis and screening of the pathways of differential metabolites, the key pathways with high correlation with metabolite differences could be found, and the results of the study showed that two metabolic pathways, Synthesis and degradation of ketone bodies and Butanoate metabolism, had a significant intervention effect (*P* < 0.05) (Figure 9). This suggests that energy metabolism is likely to be closely linked to the mechanisms by which FLN improves AF.

#### 3.3.5 Adverse events

**FIGURE 10:**
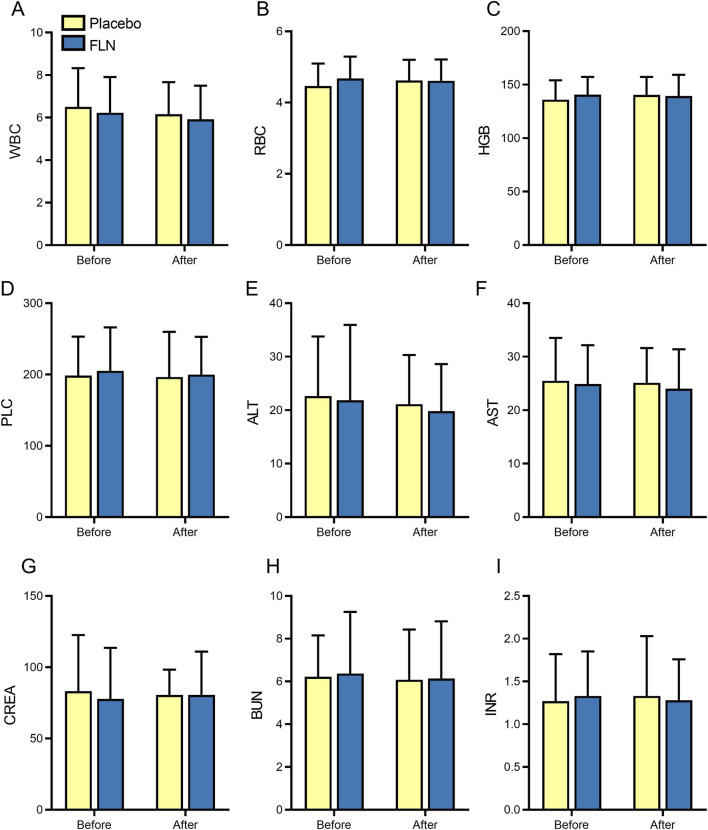
Analysis of security indicators. **(A–I)** WBC; RBC; HGB; PLC; ALT; AST; CREA; BUN; INR.

**FIGURE 11:**
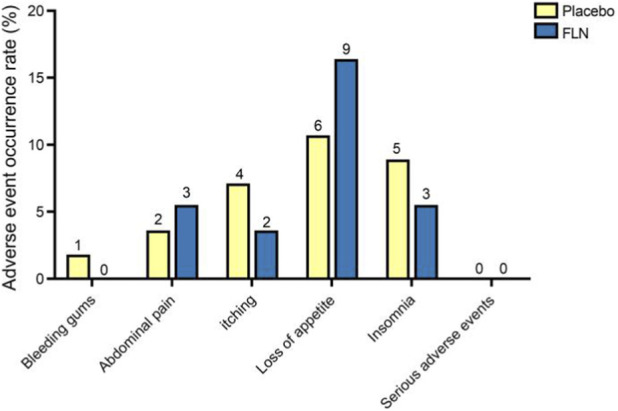
Analysis of adverse events.

Analysis of safety indicators such as blood routine, liver function, renal function, and international normalized ratio (INR) of patients in the FLN group and the placebo group before and after treatment revealed that the differences between the groups before and after treatment were not statistically significant (*P* < 0.05) (Figure 10), indicating compliance with the basic safety evaluation. No serious adverse events were reported during the study period (Figure 11).

## 4 Discussion

This trial is the first randomized, double-blind, placebo-controlled study to evaluate whether oral FLN, combined with conventional medication, can reduce the duration, frequency, and ventricular rate of symptomatic AF. Our findings indicate that FLN significantly decreased AF episodes in patients with paroxysmal AF who had an inadequate response to conventional treatment and also contributed to lower ventricular rates in AF. Additionally, FLN showed good safety and efficacy, with no significant adverse effects. The potential therapeutic benefits may be attributed to its regulation of energy metabolism.

FLN’s therapeutic potential is rooted in its sophisticated integration of TCM principles and modern pharmacological evidence. Developed from the clinical heritage of Professor Zhongxiang Lin, the formula strategically combines Shengmai San and Ganmai Dazao Decoction to achieve a synergistic effect. By addressing both root deficiencies (qi-yin insufficiency) and symptomatic manifestations (heat, stagnation, and emotional imbalance), FLN effectively restores the yin-yang equilibrium essential for managing palpitations. Clinically, FLN has demonstrated promising efficacy in treating various arrhythmias, with an annual usage of approximately 20,000 boxes at Longhua Hospital. Preclinical studies have shown that FLN effectively counteracts aconitine and calcium chloride-induced arrhythmias in rats, potentially through the inhibition of sodium and calcium ion channels (Shen et al., 2013). Further analysis divided FLN into three subgroups—Modified Shengmai San, Modified Ganmai Dazao Decoction, and a simplified FLN formula. All delayed barium chloride-induced arrhythmias and reduced their duration, showing protective effects (Xu et al., 2014). These findings suggest a strong correlation between the formula’s composition and its anti-arrhythmic properties.

In this study, we included symptomatic AF patients, regardless of whether their AF was paroxysmal or non-paroxysmal. Symptomatic AF patients often experience anxiety, depression, and reduced quality of life (Freeman et al., 2015; Heidt et al., 2016). In China, despite the availability of radiofrequency ablation, many patients refuse the procedure due to concerns about surgical trauma or side effects, while others experience AF recurrence post-ablation. Even with standardized Western medicine, AF episodes often remain difficult to control, leading many patients to seek TCM treatment. Notably, our recent study made a compelling observation: despite providing only 1 month of free medication, patients in the treatment group experienced significantly fewer AF episodes and shorter episode durations compared to the control group (*P* < 0.05) in paroxysmal AF. In patients with permanent AF, FLN also significantly reduced ventricular rates. Furthermore, the treatment group demonstrated improvements in anxiety, depression, and quality of life. These findings suggest that FLN treatment can effectively reduce AF episode frequency and duration while alleviating clinical symptoms. However, we did not observe significant improvements in cardiac function, which may require longer-term clinical follow-up for further validation.

FLN has a strong theoretical foundation supported by both its formulation principles and modern pharmacological research, demonstrating excellent efficacy in arrhythmia management. The formula contains several medicinal botanical drugs such as Kushen, Danshen, Licorice, and Huangqin, all of which have anti-arrhythmic effects (Zhang et al., 2023). The total alkaloids and flavonoids of Kushen exert a “quinidine-like” effect by modulating myocardial potassium and sodium ion channels, reducing excitability, and prolonging the refractory period, thereby inhibiting ectopic pacemaker activity (Liu et al., 2017). In a guinea pig myocardial hypertrophy model, Danshenol II-A reduces the density of fast- and slow-activating delayed rectifier potassium currents in hypertrophic myocardial cells, improving electrophysiological abnormalities (Lu et al., 2022). Licorice has a cardioprotective effect, mainly due to the flavonoid and glycyrrhizin metabolites it contains, both of which have anti-arrhythmic and myocardial cell protective effects (Wang et al., 2025; Wang et al., 2023). The pharmacological studies on these individual botanical drugs provide evidence for the efficacy of FLN in treating arrhythmias. To further explore the potential upstream mechanisms of FLN in treating AF, we conducted a non-targeted plasma metabolomics study.

Emerging evidence links metabolic disorders to AF through electrophysiological and structural remodeling (Watanabe et al., 2008). Conversely, AF itself induces metabolic remodeling, disrupting lipid and ketone metabolism, impairing mitochondrial function, and altering cardiac energy supply (David et al., 2024). Ketone metabolism plays a key role in energy compensation during AF, where high-frequency contractions deplete ATP, prompting increased ketone utilization. As ketone metabolism is more efficient, it reduces oxygen consumption and supports myocardial energy supply (Hariharan et al., 2017). Thus, targeting ketone metabolism—by inhibiting excessive utilization or regulating enzyme activity—may offer a novel therapeutic approach.

Our study suggests that the antiarrhythmic effects of FLN involve a sophisticated recalibration of the metabolic network, particularly centered on butyrate, ketone, and glycerophospholipid metabolism (Zhang et al., 2021). Butyrate undergoes β-oxidation to produce acetyl-CoA, a precursor for ketone synthesis (Ota et al., 2020), and its dysregulation correlates with increased AF risk. Following FLN treatment, we observed a significant downregulation of methyl butyrate and succinate semialdehyde (SAS). As an intermediate in the GABA shunt, the reduction of SAS likely prevents the over-accumulation of metabolic byproducts that trigger oxidative stress, while modulating acetyl-CoA levels to optimize ketone synthesis (Hariharan et al., 2017). Furthermore, the significant upregulation of acylcarnitines—including dodecanoylcarnitine, L-octanoylcarnitine, and decanoylcarnitine—suggests enhanced fatty acid β-oxidation. This is critical for AF therapy, as it indicates a restoration of metabolic flexibility, shifting the heart back toward efficient energy substrates and increasing acetyl-CoA availability for regulated ketone production (Sun et al., 2025). Beyond energy supply, FLN demonstrated a profound impact on protective metabolites. The marked increase in Lutein (a potent antioxidant) and Biliverdin (a cytoprotective agent) suggests that FLN suppresses AF by scavenging reactive oxygen species and mitigating inflammation. Additionally, the enrichment of glycerophospholipid metabolism, evidenced by the upregulation of LysoPC (22:0), points toward the stabilization of the atrial cardiomyocyte membrane, which is essential for preventing electrophysiological leakage and structural remodeling.

In conclusion, these findings indicate that the key metabolites identified in our profiling work regulate AF by integrating energy supply optimization, antioxidant defense, and membrane stabilization. Further exploration of these coordinated mechanisms will provide deeper theoretical support for the clinical application of FLN in AF management.

## 5 Limitations

This study has several limitations. First, the COVID-19 pandemic posed challenges to conducting regular Holter follow-ups and limited patient enrollment, particularly affecting the scalability of a multicenter trial. Second, FLN contains multiple bioactive metabolite botanical drugs whose therapeutic benefits likely arise from synergistic effects. Although a preliminary metabolomic analysis was conducted, further mechanistic studies are warranted to fully elucidate its mode of action.

## 6 Conclusion

In conclusion, this randomized clinical trial found that FLN treatment improved the frequency and duration of AF episodes, reduced ventricular rate, and alleviated clinical symptoms in symptomatic AF patients. This study also used metabolomics to explore FLN’s role in AF treatment, providing new insights into TCM-based management.

## Data Availability

The raw data supporting the conclusions of this article will be made available by the authors, without undue reservation.
