# Supplementary material for: Clinical efficacy and mechanistic study of fulvning granules in symptomatic atrial fibrillation: a randomized controlled trial with untargeted metabolomics analysis
Source: Front Pharmacol. 2026 Feb 24;17:1761563. doi: 10.3389/fphar.2026.1761563 (PMC12971955; doi:10.3389/fphar.2026.1761563)
Supplement: Supplementary file 1 [file Supplementaryfile1.docx]

**Supplementary Table1**

Mobile phase gradient

| Time (min) | A% | B% |
| --- | --- | --- |
| 0-8 | 0-20 | 100-80 |
| 8-20 | 20-28 | 80-72 |
| 20-28 | 28-65 | 72-35 |
| 28-32 | 65-95 | 35-5 |
| 32-35 | 95 | 5 |
| 35-35.1 | 95-0 | 5-100 |
| 35.1-38 | 0 | 100 |
