# Supplementary material for: Clinical efficacy and mechanistic study of fulvning granules in symptomatic atrial fibrillation: a randomized controlled trial with untargeted metabolomics analysis
Source: Front Pharmacol. 2026 Feb 24;17:1761563. doi: 10.3389/fphar.2026.1761563 (PMC12971955; doi:10.3389/fphar.2026.1761563)
Supplement: Supplementary file 2 [file Supplementaryfile2.docx]

**Supplementary Table 2**

UPLC-Q-TOF-MS Identification of Main Compounds in FLN

| **No** | **Time（min**） | **Name** | **Ion mode** | **Mass calculation error(ppm)** | **Molecular formula** | MS/MS data | **Compound Type** | **CAS** | **Peak Area** |
| --- | --- | --- | --- | --- | --- | --- | --- | --- | --- |
| 1 | 0.96 | Betaine | [M+H]^+^ | 2.5 | C_5_H_11_NO_2_ | 118.0854;58.0643 | Alkaloid | 107-43-7 | 108817 |
| 2 | 4.46 | Matrine | [M+H]^+^ | 4.0 | C_15_H_24_N_2_O | 249.1955;176.1061;150.1272;148.1115 | Alkaloid | 519-02-8 | 9184722 |
| 3 | 5.23 | Danshensu | [M-H]^-^ | 2.0 | C_9_H_10_O_5_ | 179.0362;135.0458;123.0456 | Phenolic | 76822-21-4 | 502379 |
| 4 | 5.64 | Piscidic acid | [M-H]^-^ | 4.3 | C_11_H_12_O_7_ | 255.0508;193.0512;179.0351;165.0559;133.0296;107.0501;72.9932 | Phenolic | 469-65-8 | 1813026 |
| 5 | 10.47 | Chrysin-6-C-arabinoside-8-C -glucoside | [M-H]^-^ | 2.4 | C_26_H_28_O_13_ | 547.1478;487.1245;457.1143;427.1036;367.0817;337.0697 | Flavonoid | 185145-33-9 | 2658566 |
| 6 | 10.54 | Liquiritin | [M-H]^-^ | -1.0 | C_21_H_22_O_9_ | 417.1207;255.0666;135.0091;119.0505 | Flavonoid | 551-15-5 | 454246 |
| 7 | 10.73 | Hyperoside | [M-H]^-^ | 1.3 | C_21_H_20_O_12_ | 463.0918;301.0358;300.0263;271.0239 | Flavonoid | 482-36-0 | 122780 |
| 8 | 11.06 | Chrysin 6-C-glucoside-8-C-arabinoside | [M-H]^-^ | 3.3 | C_26_H_28_O_13_ | 547.1476;457.1152;427.1050;367.0817;337.0711 | Flavonoid | 185145-34-0 | 1804317 |
| 9 | 13.35 | Lobetyolin | [M+FA-H]^-^ | 2.7 | C_20_H_28_O_8_ | 441.1672;215.1093;185.08967;159.0815;143.0703 | Other | 129277-38-9 | 58333 |
| 10 | 15.30 | Baicalin | [2M-H]^-^ | -0.3 | C_21_H_18_O_11_ | 445.0791;269.0454;175.0254 | Flavonoid | 21967-41-9 | 13229448 |
| 11 | 15.93 | Salvianolic acid B | [M-H]^-^ | 2.9 | C_36_H_30_O_16_ | 717.1517;519.0970;339.0526;321.0400;295.0613 | Phenolic | 121521-90-2 | 3214742 |
| 12 | 17.42 | Norwogonin 7-O-β-D-glucuronide | [M-H]^-^ | 3.8 | C_21_H_18_O_11_ | 445.0755;269.0451;164.9826 | Flavonoid | 119152-50-0 | 2700017 |
| 13 | 17.80 | Salvianolic acid A | [M-H]^-^ | -0.4 | C_26_H_22_O_10_ | 493.1118;295.0600;185.0237;159.0444 | Phenolic | 96574-01-5 | 1595675 |
| 14 | 18.47 | Chrysin-7-O-glucuronide | [M-H]^-^ | 1.4 | C_21_H_18_O_10_ | 429.0834;253.0509;209.0608;175.0245 | Flavonoid | 35775-49-6 | 1300158 |
| 15 | 18.71 | Wogonoside | [M-H]^-^ | 4.6 | C_22_H_20_O_11_ | 283.0604;268.0365;175.0239;113.0243 | Flavonoid | 51059-44-0 | 3473438 |
| 16 | 19.93 | Oroxylin A-7-O-glucuronide | [M-H]^-^ | 0.7 | C_22_H_20_O_11_ | 283.0601;268.0369;175.0247;163.0038 | Flavonoid | 36948-76-2 | 6600557 |
| 17 | 23.16 | Baicalein | [M-H]^-^ | -0.7 | C_15_H_10_O_5_ | 269.0458;251.0359;223.0405;195.0459;169.0647;136.0987 | Flavonoid | 491-67-8 | 1488525 |
| 18 | 25.92 | Glycyrrhizic acid | [M-H]^-^ | 2.3 | C_42_H_62_O_16_ | 821.3948;351.0565 | Triterpenoid saponin | 1405-86-3 | 1900811 |
| 19 | 26.04 | Wogonin | [M-H]^-^ | -1.4 | C_16_H_12_O_5_ | 283.0621;268.0372;240.0434;211.0378;163.0029 | Flavonoid | 632-85-9 | 1304916 |
| 20 | 26.48 | Skullcapflavone II | [M-H]^-^ | -1.9 | C_19_H_18_O_8_ | 373.0940;358.0705;343.0463;328.0230;300.0280 | Flavonoid | 55084-08-7 | 505157 |
| 21 | 26.66 | Schisandrol A | [M+H]^+^ | 0.2 | C_24_H_32_O_7_ | 433.1912;384.1923;369.1694;353.1743;338.1515 | Phenylpropanoid | 61281-38-7 | 221301 |
| 22 | 27.68 | Schisandrol B | [M-H_2_O+H]^+^ | 0.5 | C_23_H_28_O_7_ | 399.1805;368.1541;353.1379;337.1393;330.1115 | Phenylpropanoid | 58546-54-6 | 80970 |
| 23 | 30.11 | Cryptotanshinone | [M+H]^+^ | 4.0 | C_19_H_20_O_3_ | 297.1499;279.1393;254.0943;251.1448 | Diterpene | 35825-57-1 | 41793 |
